# Illegal Trade in Protected Sharks: The Case of Artisanal Whale Shark Meat Fisheries in Java, Indonesia

**DOI:** 10.3390/ani13162656

**Published:** 2023-08-17

**Authors:** Vincent Nijman

**Affiliations:** Oxford Wildlife Trade Research Group, School of Law and Social Sciences, Oxford Brookes University, Oxford OX3 0BP, UK; vnijman@brookes.ac.uk

**Keywords:** CMS, illegal unreported and unregulated fishing, *Rhincodon typus*, Southeast Asia, wildlife trade

## Abstract

**Simple Summary:**

Illegal fishing, including that of sharks for meat and fins, is one of the larger threats to marine biodiversity conservation. Getting data on these illegal activities is challenging as there are few reliable official records. I use data from the media, tourists, and artisan fishermen to gain insight into the trade in the world’s largest fish, the whale shark, in Indonesia. Whale sharks are typically caught in fishing nets, dragged alongside boats to the shallows, where they are butchered. The meat and oil are sold. In a popular tourist area, Pangandaran, whale sharks are landed and butchered on the beach in view of hundreds of people and local media; I report on 30 landings (2002–2022). Along the south coast of Java, part of which includes Pangandaran, I document 38 landings (2019–2022). Artisanal fishermen see the landings of whale sharks as fortuitous events, and the monetary gains are frequently shared with the community. However, artisanal fisheries pose a significant threat to whale sharks, and the legal protection that whale sharks receive in Indonesia is not sufficiently enforced. Furthermore, Indonesia is a signatory to various international agreements that preclude the fishing and trade in whale sharks, and greater adherence to these rules and regulations is needed.

**Abstract:**

Illegal, unreported, and unregulated fishing, including that of sharks, poses a significant threat to marine ecosystems and individual species. I use data from the media, tourists, and artisan fishermen to gain insight into the trade in the world’s largest fish, the whale shark (*Rhincodon typus*). I focus on the Indonesian island of Java where, along its south coast, whale sharks are landed and butchered on the beach in view of hundreds of people and local media. Whale sharks are typically caught in fishing nets and dragged alongside boats to the shallows, where they are butchered. The meat and oil (valued at ~USD 2000 per shark) are sold and distributed within the community. I document 58 landings of mainly immature whale sharks (2002–2022). Artisanal fishermen see the landing of whale sharks as a fortuitous event, but the species is protected, and Indonesia is a signatory to various international agreements that preclude the fishing of whale sharks. It is imperative for the conservation of whale sharks that the various parties in Indonesia adhere better to their own rules and regulations protecting this species.

## 1. Introduction

Illegal, unreported, and unregulated fishing is one of the greatest threats to marine ecosystems. It undermines efforts to sustainably manage fisheries and harms the conservation of marine biodiversity. While it is challenging to estimate the global extent of illegal fisheries, one often cited but now somewhat dated study puts its value at USD 10–23.5 billion per year^−1^ [1]. One of the fisheries that is often brought up with regard to illegal fisheries is the fishing of, and subsequent trade in, sharks [2].

The global shark trade is largely driven by the demand for shark fins, which are consumed as a traditional and socially important luxury food item [3,4]. Over the last decades, the expanding consumer purchasing power in especially eastern Asia has led to an increase in shark fin trade and has put increasing pressure on many shark species. Recent research in one of the major shark fin trade centres in Hong Kong, for instance, found that two-thirds of the species on offer were globally threatened [5]. Several species of shark are vulnerable to overexploitation and unsustainable fishing because of their large size, slow life histories, and the high monetary value of easy-to-preserve parts (fins, cartilage, etc.).

Globally, Indonesia is the main exporter of sharks and shark fins, of around 100,000 tonnes a year since the year 2000 [6]. There is uncertainty about how much of the shark catch in Indonesian waters is taken as bycatch of fisheries (using longlines, driftnets, handlines, and purse seines), how much of the fisheries is targeted specifically at sharks, and to what extent shark fisheries contribute to the livelihood for many artisanal fishers [7,8]. International policy responses to the negative effects of shark fishing have resulted in the implementation of shark finning bans in some areas, whereas others focused on promoting the full use of dead sharks such as discouraging carcass discards after defining [3]. Starting roughly ten years ago, the Indonesian authorities implemented a series of measures to better regulate the domestic and international trade in sharks in and from Indonesia. The shark species that are listed on one of the appendices of the Convention on International Trade in Endangered Species of Wild Fauna and Flora (CITES) are either precluded from being traded commercially, or there are regulations in place for their export. Much of this regulation concerns species that are traded internationally, and, indeed, most are implemented and enforced at the point of export. The effect of protective measures, fishing restrictions, and export regulations may have a limited effect on local demand or on artisanal fisheries in general.

The UN Sustainable Development Goal 14 (“Life below water”) aims to conserve and sustainably use the oceans, seas, and marine resources, and within this, target 14 b works to provide access rights to artisanal fishers on marine resources and markets. Small-scale and artisanal fishers tend to fish in areas close to the coast and within the exclusive economic zone of a country. Access to key international markets is often challenging, leading to much of the sales occurring at local markets [9]. Globally, a number of assessments have been made of artisanal shark fishing [10,11,12,13], evaluating the extent to which sharks contribute to the livelihoods of artisanal fishing communities. The information we have on this from Indonesia is limited (but for some earlier work, see [14,15]).

The whale shark (*Rhincodon typus*) is the largest species of fish; a 4-year-old individual would have reached a length of ~4 m, whereas adult females can reach lengths of ~15 m or more. It is a highly migratory epipelagic and neritic species with a circumglobally distribution in all tropical and temperate waters [16]. Movements are linked to localised planktonic blooms and water temperature changes.

Artisanal fishing for whale sharks occurs in a limited number of countries, including Iran, Pakistan, the Maldives, Indonesia, and the Philippines [16,17,18], and more large-scale commercial fishing occurs (or did occur) in India, China, and Taiwan [19,20,21,22]. The species is listed as Endangered on the IUCN Red List, and especially the Indo-Pacific subpopulation has seen a drastic decline [17]. To better manage and protect the remaining populations, whale sharks are included in Appendix II of CITES, Appendix II of the Convention of Migratory Species of Wild Animals (CMS) and Annex I (highly migratory species) of the UN Convention on the Law of the Sea (UNCLOS). Accidental whale shark catches are also covered as part of the Convention for the Conservation and Management of Highly Migratory Fish Stocks in the Western and Central Pacific Ocean (WCPFC Convention). Indonesia is a Party to CITES (signed 1978, ratified 1979), UNCLOS (signed 1994, ratified 2000) and the WCPFC Convention (signed 2001, ratified 2013). The country is not a Party to the CMS, but it has signed several Memoranda of Understanding with the CMS.

Whale sharks’ protection in Indonesia is covered in the Fisheries Law; it is unlawful to ship, distribute or keep fish that inflict financial costs on the community, or to fish without a fishing license. Penalties can include fines of up to USD 106 606 and/or six years in prison (maximum penalties for artisanal fishermen, *nelayan kecil*, are less). In 2013, the species was included on Indonesia’s protected species list (most recently updated as Regulation No. 20 of the 2018 Ministry of Environment and Forestry). As a protected species, it is illegal to capture, transport, sell, or buy whale sharks, and fines of up to USD 7107 and/or a five-year prison sentence can be imposed on lawbreakers. Prior to 2013, commercial trade in the species was not permitted either, as no harvest quota had been allocated for the species (in Indonesia, all non-protected CITES-listed species do require a harvest quota if one wishes to trade in them). Despite national protective measures and international commitments, there are occasional reports of whale sharks being caught and traded within Indonesia [18,23,24].

Here, I report on the landings of whale sharks by artisanal fishers in Indonesia, and the subsequent trade in whale shark parts. Specifically, I address (1) the number of landings in Pangandaran Bay, and the south coast of Java in general; (2) the temporal distribution (i.e., months of the year) of the landings of whale sharks; (3) the size (and inferred age) of whale sharks that are landed along Java’s south coast; (4) the views that artisanal fishermen have of whale sharks and the monetary value they represent to the fishing community.

## 2. Materials and Methods

### 2.1. Study Area: Pangandaran and Java’s South Coast

**Figure 1:**
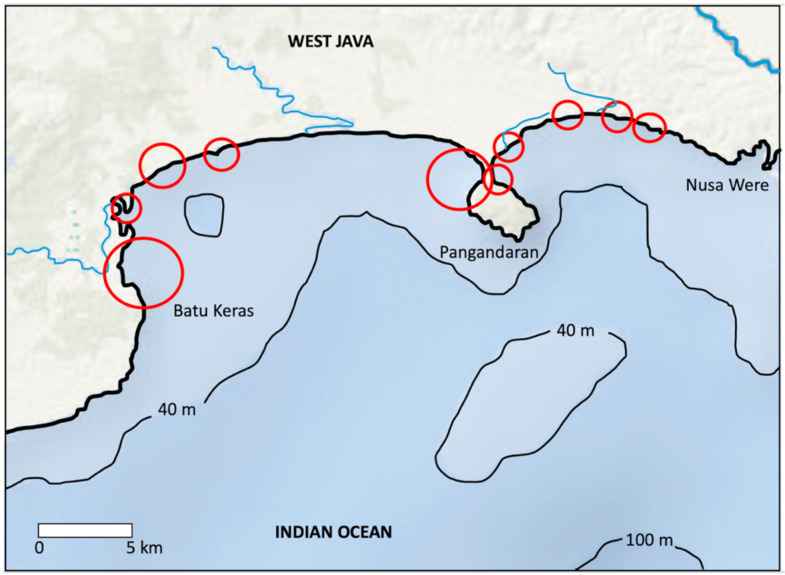
Study area: Pangandaran Bay on the south coast of West Java, Indonesia, with records of whale shark landing (red circles) and trade over the period 2002–2020. Size is proportional to the number of records.

I focus on Pangandaran Bay (Teluk Pangandaran and parts of Teluk Panajung Barat) covering 40 km of coastline, between Batu Karas in the west and Nusa Were in the east, representing the southeasternmost part of the province of West Java (Figure 1).

This stretch of coastline used to be part of the Ciamis Regency, but in 2012, this regency was split into two, creating the Pangandaran Regency in the south. Prior to 2012, Pangandaran referred to the Pangandaran peninsula only (the remainder was Ciamis), whereas, after that, it referred to either the peninsula or the regency as a whole.

There are four formal landing places or harbours (*darmaga*/*pelabuan nelayan*) in Pangandaran Bay, from west to east, Batukeras, Bojong Salawe, Pantai Timur Pangandaran, and Cikidang, in addition to smaller landing sites on beaches and sheltered bays. Based on my own observations, I estimate that about 150 fishing boats are active in Pangandaran Bay, about half of them in Pangandaran and Cikidang. Most are small boats that only go out on day trips, venturing five to eight km out at sea. In 2019, 3.8 million tourists, over 99% from within Indonesia, visited the Pangandaran Regency [25]; even in the least visited months (February and May), around 100,000 tourists were still present.

While shark fishing and trade is common in Pangandaran village, including substantial numbers of hammerhead sharks ([26]; V. Nijman, pers. observ.), it has been rarely singled out as an important shark fin trade port. Locally, shark meat is sold and consumed as salted fish (*ikan asin*) [26,27], and shark oil (*minyak hiu*) is sold in small bottles as aphrodisiacs. Despite an absence of records from the Indian Ocean for their modelling, Morales-Ramirez and Wang [28] found the area just south of Pangandaran Bay to be highly suitable for whale sharks. One of twenty-nine whale sharks tagged at Ningaloo Reef, western Australia, migrated to Java’s south coast and spent time southeast of Pangandaran Bay [29], demonstrating that the whale sharks in Pangandaran Bay are part of a larger Indian Ocean population. This all suggests that Pangandaran Bay may have an important role to play in the conservation of whale sharks, both in an Indonesian and a wider Indian Ocean setting. In a broader context, Java has been recognised for some importance to the whale shark, especially the area around Probolinggo, on the north coast of East Java, where whale sharks seasonally congregate and where they can be easily observed [30].

### 2.2. Data Collection in Pangandaran Bay

Between 1995 and 2019, I made 12 visits to the region, including to Pangandaran, Karangnini, and Batu Karas. Each visit lasted between two and five days, totalling 36 days. The visits were spread out over the year, with four visits from December to February, one from March to May, four from June to August and three from September to October. I used a mixed approach in that data were collected both quantitatively and qualitatively, and both are used here to provide a narrative of whale shark landings and trade. In the first years, data collection was focused on general natural resource management and was largely qualitative, whereas in more recent years (2012 onwards), when I had become more familiar with the area and the community, it was more focused on specific taxa including certain rays and sharks.

While in Pangandaran Bay, I had discussions and conversations with artisanal fishermen, officers of the nature reserve, police officers, and representatives of the fisheries department. In later years, people recognised me from earlier visits and rapport was quickly re-established, allowing conversations to be open and frank. Conversations often started with a focus on marine turtles and large marine molluscs (despite their protected status, these species are traded widely and openly [31,32]) and then moved on to other large animals such as manta rays (*Mobula* spp.) and whale sharks. For whale sharks, I asked for details about landing or stranding (both referred to here as landings; see below), if any, and people’s perception of whale sharks and their economic importance. Whale sharks in Pangandaran Bay are referred to as *hiu naga* (translated as dragon shark) or *hiu naga bintang* (starred dragon shark), occasionally as *hiu tutul* (spotted shark), *hiu besar/gede* (large shark) or *hiu bodoh* (stupid shark); in my experience, only government officials and sometimes the national media use the word *hiu paus* (whale shark).

Whale shark fishing in Pangandaran Bay involves several hours of butchering on the beach or in the shallows and is therefore noticed by many other than the fishermen that made the catch. As such, there were many options to cross-reference observations while in Pangandaran Bay. In addition, I was able to cross-reference observations with records in the Indonesian media (television, newspapers, and blogs).

### 2.3. Data Collection of Other Coastal Areas of Java and Perceptions towards Whale Sharks

Over the same period that I stayed in Pangandaran Bay, i.e., between 1995 and 2019, I visited several other coastal regions of Java. On the south coast, this included Ujung Kulon (in the province of Banten), Palabuan Ratu (West Java), Nusakambangan and Segara Anakan (Central Java), Parangtritis (Yogyakarta), and Balekambang and Sukamade (East Java). On the west, north, and east coasts, this included Anjer (Banten), Pekalongan, Tirang-Semarang (Central Java), Wonorejo, Sumenep, Pasir Putih, Baluran, and Walu Dodol (East Java). Between August 2019 and February 2023, I searched the Internet for records of landings or strandings of whale sharks using the Indonesian words for whale shark as search terms (see above). From the various reports, I extracted information on the exact location, date, and size of the whale shark (in metres, since data on mass were deemed unreliable), and the fate of the animal (e.g., pushed back to sea, buried, butchered, or sold). Many reports were accompanied by photographs or videos taken by tourists or the local media, typically with both the whale shark and people in the same frame. This allowed for the verification of species and size.

From the discussions I had with residents and fishermen in Pangandaran, from the information presented in news stories, and from comments posted on social media accompanying the videos or photos of whale shark landings, I extracted information on the perceptions of people towards whale sharks. From the information provided by fishermen and the timing of the landings, I extracted temporal information on the presence of whale sharks along Java’s south coast. All translations from Bahasa Indonesia (Indonesian, spoken throughout Indonesia) or Basa Sunda (Sundanese, spoken in western Java) are mine. In English, landing would refer to a whale shark being actively moved onto the beach or into the shallows by fishermen using nets or lines for instance, whereas stranding would indicate the shark ends up on the beach in a passive manner, accidentally, by its own accord. While this difference is also apparent in Bahasa Indonesia (landing = *terjerat* or *digiring*; stranding = *terdampar*) and Basa Sunda (landing = *kajiret* or *giring*; stranding = *kapalidkeun*), in practice, these terms are used interchangeably, and here I will refer to them as landings.

### 2.4. Analysis and Estimates of the Total Number of Whale Sharks Landed

I enlarged photographs of landed whale sharks to around 1:20 and measured the total length (tip of the nose to tip of the tail), and converted this to the nearest 0.5 m, assuming an average Indonesian man has a height of 1.70 cm (most photographs or videos of landings had Indonesian men included). Following [22] this, I assumed that meat comprises 45% of the body weight of a whale shark, and I used the lower-end value for shark meat in Pangandaran of USD 1.80 kg^−1^ to estimate the monetary values [26,33]. Prices were reported in Indonesian rupiah; I corrected these for inflation to December 2022 and then converted this to US dollars.

Whale shark landings do not occur in a predictable manner or at a predictable time, and in terms of detecting it, I was dependent on whether others (fishermen, tourists, or local media) announced this; hence, by default, the data collection was somewhat opportunistic. This also means that it is certain that I was not able to detect and document all landings. Hence, any analysis is based on a subset of what was landed, and the numbers I present are thus minimum estimates. That said, to gain a better understanding of the total number of whale sharks that may be landed along Java’s south coast on an annual basis, I used two complementary approaches. One assumes that data from the last 14 years from Pangadaran Bay and its ports of Cikidang and Bojong Salawe, are representative of nine or ten other such fishing areas along the south coast (there are at least 18 other fishing ports the size of Cikidang and Bojong Salawe or larger along Java’s south coast). It is difficult to extrapolate the findings from Pangandaran Bay to Java’s north coast, as this is a much shallower coast with a very different likelihood of landings of whale sharks. For the second approach, I focus on the most recent period (August 2019 to December 2022) and the number of whale sharks that are landed along all of Java’s coast as reported in the Indonesian media over this period—acknowledging that many of these may not be reported on.

For statistical analysis, data were log-transformed prior to testing so as to approach a normal distribution more closely. Tests were run in SocSciStatistics/2023, and significance was accepted when *p* < 0.05 in a two-tailed test. I present means ± s.d.

## 3. Results

### 3.1. Landings in Pangandaran Bay

I document 30 landings of whale sharks in the 40 km-long Pangandaran Bay (Table 1). A total of 29 of these were recorded in the last 14 years (2009–2022). There were no records for April, May and October, and there were two temporal peaks in landings, a main one from June to September and a smaller one from November to February. For the whale sharks for which we had the total length reported in the media, and for which I could measure from photographs or video stills, there was a very strong correlation between the estimated and reported length (Pearson’s R = 0.953, R^2^ = 0.908, *p* = 0.00002). For the 15 whale sharks for which we either had a reported or a measured total length, the mean was 6.9 ± 2.9 m (range 3.5–15; median size was 6.7 m).

### 3.2. Landings of Whale Sharks along Java’s South Coast

Table 2 lists the recent landings of whale sharks along Java’s south coast, many of which were reported in a wide range of media (newspapers, online news reports, television, blogs, vlogs, and other social media). The peak of landings was between July and October, with single records in February, March, April, and November.

**Figure 2:**
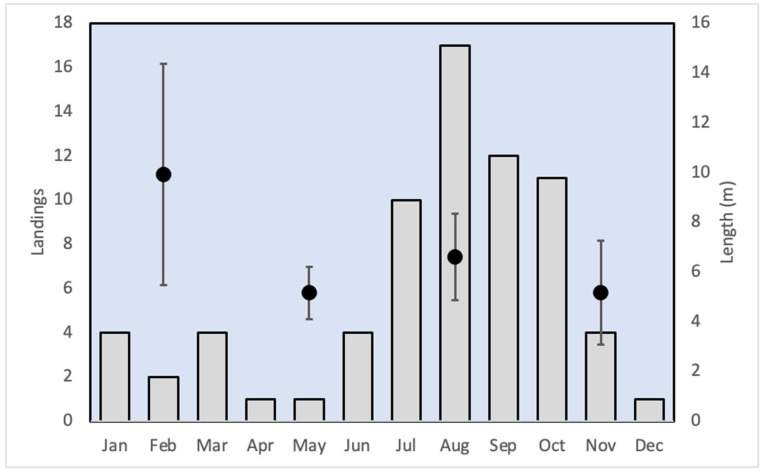
Temporal distribution of 71 whale shark landings on Java (bars), and their reported or estimated length in meters (mean ± s.d.) for four 3-month periods (circles). Data from this study and [34,35].

Combining all the records (i.e., from Pangandaran Bay and the remainder of Java’s coast), the mean reported length of whale sharks landed along Java’s coast was 6.7 ± 2.5 m (median size was 6.0 m). Assuming an equal temporal distribution of landings, the months of April and May had fewer landings, and the months of August and September had more whale shark landings than the other months combined (χ^2^ = 6.51, df = 1, *p* = 0.011 and χ^2^ = 22.48, df = 1, *p* = 0.00001, for April–May and August–September, respectively). There was a tendency for whale sharks that landed in the early months of the year to be somewhat larger (but still mostly not adults) than ones that were landed in other parts of the year, but the difference was not statistically significant (One-way Anova, F_1,20_ = 0.9776, *p* = 0.393) (Figure 2). Six large, and presumably adult, individuals, of between an estimated length of 8.5 to 15 m, were landed in January (1), August (3), October (1) and November (1), thus matching the overall temporal pattern of landings.

### 3.3. Estimates of the Number of Whales Landed on Java’s South Coast

**Figure 3:**
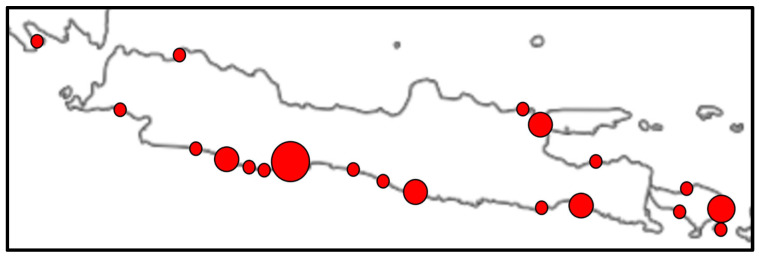
Landings of whale sharks in and around Java, Indonesia, between 2012 and 2022 (red circles), based on this study and [34,35] sizes are roughly proportional to the number of landings. The largest circle is Pangandaran Bay (see Figure 1).

A minimum of 29 whale shark landings in Pangandaran Bay over the last 14 years equates to over two whale sharks a year. The largest number of annual landings was 5 (in 2009) and 6 (in 2021) and in four years, no landing was reported (2012, 2014, 2017, and 2019). If the years with no landings were due to under-reporting and we focus on years with at least one report, the mean number approaches three whale sharks landed a year. With at least nine or ten other comparable harbour and port areas along Java’s south coast, and while it is challenging to scale up landings across the region, as a guestimate, this may suggest that perhaps 15 to 30 whale sharks are landed per year. If the total number of recent reports along Java’s south coast is an accurate reflection of the true number of landings (Table 2), this gives a lower estimate of a minimum of 11 whale sharks landed per year. The difference between the two is most likely because of a higher detection and reporting rate of whale shark landings in Pangandaran (because of the presence of many tourists) than at other, more remote, parts of Java’s south coast. This again underscores the assumption that what is reported here are minimum numbers. Finally, there are distinctly more landings along Java’s south coast than along its northern coastline (Figure 3) and including the ones that do land along the north coast would add to the total.

### 3.4. Views on Whale Sharks and Their Monetary Value

**Figure 4:**
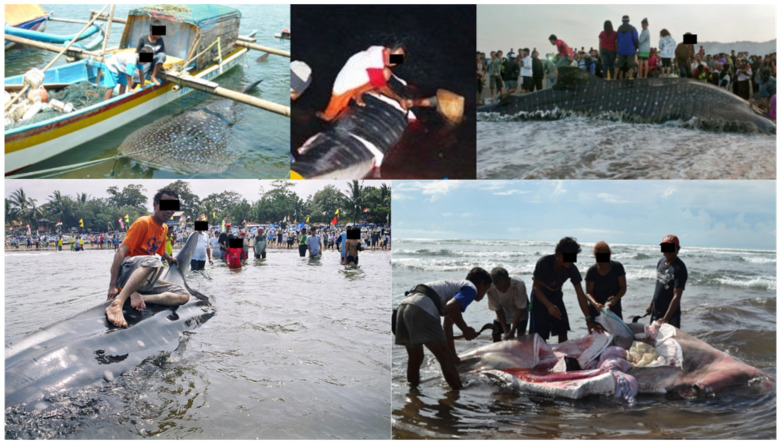
Whale sharks landed at Pangandaran Bay. From top left, clockwise: caught and attached to a fishing boat in Karangjaladri, Sep. 2013; butchering at night, Pangandaran, Jan. 2011; tourists standing on and around a landed whale shark, West Beach, Nov. 2016; butchering of whale shark in the shallows, Babakan, Nov. 2016; tourist sitting on whale shark, East Beach, Sep. 2002.

Fishermen in general have mixed feelings towards whale sharks. On one hand, they are seen as large, sometimes stupid, fish that can do a lot of damage to their nets. There is a general awareness that the species is not one that can be traded easily because of its protected status. On the other hand, once a whale shark is landed, it is seen as a positive sign in two different ways. Firstly, the meat, the liver (oil), and the fins (although these are rarely singled out, see below) provide a source of protein for the local community, and often much-needed revenue. Secondly, the landing of a whale shark is seen as an important and reliable indicator that better times are coming for the fishermen. While it is generally well-known, both to fishermen, others in the local community, and government officials that whale sharks are a protected species, the trade in their meat occurs in the open (Figure 4).

Once a whale shark is landed, there are three options for what to do with the animal, namely (1) pushing, pulling or allowing it to go back to the sea; (2) waiting until it dies, and leaving it on the beach, burying it on the beach or dragging it out to sea; (3) butchering it, either when still alive or just after it has died. The first option is often not feasible and often illogical, given the efforts that have been made to get the animal on the beach or in the shallows in the first place. The second option is undesirable (with many referring to the smell of a rotting carcass) or requires substantial manpower. The third option is the preferred one, at least in some regions, both by the fishermen and the authorities.

I have spoken to several police officers, officers from the nature conservation agency, and fisheries inspectors that indicated that once the animal was landed, it may as well be used (a similar way of reasoning is used to justify the butchering of manta rays). The emphasis is invariably put on the meat and not on the fins, presumably as the fins are intended for export and sold to outsiders, and it is seen as a sensitive subject to talk about openly. Alternatively, if there is no access to outside markets, the shark fins actually have limited monetary value, and this is the reason why their trade is not discussed.

A conservative estimate for the mass of a 6.7 m long whale shark is ~2250 kg [19]. This may provide some 1000 kg of meat, at a market value of USD 1800. Even if only half of the meat enters the commercial trade, with the other half distributed to members of the community, the value of the meat is substantial (the government recommended monthly minimum wage for Pangandaran in 2022 was USD 132). The oil from the liver and the fins add substantially to the monetary value. Several reports indicate that the money generated by the sale of whale shark meat is distributed to the community, used for the repair of the local mosque, or for other community causes.

## 4. Discussion

### 4.1. Whale Shark Landings in Pangandaran Bay and Java

The whale shark was described based on a 4.5 m juvenile caught by fishermen in Table Bay, South Africa in 1848 [36], but it took more than 70 years for the first whale shark to be observed in the Indonesian archipelago, off southeastern Sulawesi [37]. A few years later, in 1907, Van Kampen [38] reported on two whale sharks caught along the north coast of Java, the first one was observed in the fish market in Jakarta, and the second in Surabaya harbour. Since then, published information on whale sharks in and around Java, and especially any landings of whale sharks here, is scarce; a recent review of global shark landings listed only two events for all of Indonesia [39].

The whale sharks that were documented to have been landed in Pangandaran Bay over the last two decades give important insight into both the whale sharks and the artisanal fishery communities. Contrary to Maruanaya [40], who reported that whale sharks were only present in Pangandaran in the months of August and September, the records suggest their year-round presence. The whale sharks that were landed in Pangandaran Bay were between 3.5 and 15 m in length, averaging 6.9 m, and the larger sample averaged 6.7 m. This is markedly larger than the mean of 4.3 ± 1.0 m (range 3–7 m) of live whale sharks in Indonesian waters, as reported by Himawan et al. [41]. Hsu et al. [19] estimated that male whale sharks in the Indo-Pacific Oceans begin maturing at a total length of ~8.5 m and females at a total length of ~9.5 m. Colman [42] indicated that the whale sharks of both sexes at Ningaloo Reef, western Australia, mature at more than 9 m. This then suggests that all but four of the whale sharks landed on Java were not yet adults.

The timing of the landings, and reference made by fishermen to the seasonal occurrence of these landings, may suggest that a proportion of the whale sharks are migratory, and hence, are only present on Java’s south coast for parts of the year (i.e., in the austral winter). Alternatively, and not mutually exclusive, during the austral winter, it is easier to catch whale sharks.

There is very limited data on the landings of whale sharks in Indonesia, and the data from Java fill an important gap. Reynolds et al. [28] noted that, amongst other areas, the coast of southern Java where whale sharks overlap with fishing activity, they may be at risk from opportunistic harvesting, entanglements with gear, and collisions with vessels. White and Cavanagh [18] reported on whale shark landings by artisanal fishermen from throughout Indonesia. Over a 4.5-year period, 270 spot checks at individual landing sites resulted in the record of a single whale shark caudal fin being sold in southern Bali. The shark was said to have been opportunistically caught by a haul net. After being brought alongside the small boat, the whale shark was finned, and the carcass discarded at sea. Based on information from the same trader, four more whale sharks were caught, finned and the carcasses left at sea, and discussions with shark fin processors and researchers revealed information about five other whale shark landings over the same period [18]. These records were all from between April and October [18]. Widjajanti [35] reported on the landing of three whale sharks (estimated to be 5, 7, and 8 m in length), in October 2013, at Kenjeran Beach, East Java that were sold to a local trader for USD 443. Nugraha et al. [34] reported on 17 landings of whale sharks from throughout Indonesia for the period 2012–2017, ten of them on the coasts of Java, but the data from Pangandaran Bay suggests that by no means is this a complete picture.

With regard to whale sharks, it is important to note that there is nothing special about Pangandaran Bay—there are very few sightings reported for the species (e.g., none in [34] or [28]), in size, its fishing fleet is typical for western Indonesia, and the area is not featured as an important shark fin trade hub. There is no indication that whale sharks are specifically targeted (that is, no boats set out to catch whale sharks), but they are either landed after an accidental catch or a chance encounter at sea led to it being caught and landed. While Morales-Ramirez and Wang [28] found the Indian Ocean south and southeast of Pangandaran Bay to be highly suitable for whale sharks, so were the seas surrounding Batam, north Sulawesi, Bali, Lombok, and the Lesser Sunda Islands (areas in eastern Indonesia were not included in their models). What is special about Pangandaran Bay, however, is the number of tourists that visit it. Despite its small size, this equals almost a quarter of those that visit the tourist hotspot of Bali (3.8 vs. 16.8 million for 2019). Given that most of the Pangandaran Bay tourists are from within Indonesia (primarily Java, in 2019, home to 150.4 million people), internationally the tourism attraction of Pangandaran is rarely noted. Nijman [25] estimated for 2019 that on average ~23,000 to 44,000 people (weekday/weekend) people were present on the Pangandaran peninsula each day. With so many people around, it is near-impossible for whale sharks that are landed on the beach or shallows not to be noticed. The size of the shark, the excitement the landing generates, and the fact that it happens only a few times a year, make it newsworthy enough for it to be covered by the local media; in addition, many tourists will post photographs of the event on social media. The use of the public in obtaining data on whale sharks (‘citizen science’) has been used to obtain data on migration patterns and population parameters [43,44], their global distribution, migration, and size distributions [45], and even to report a decline in the number of whale sharks that were killed [46]. However, until this research, to the best of my knowledge, it has not been used to gain insight into the killing and trade in whale sharks [47].

If the estimate of 10 to perhaps 30 whale shark landings per year along Java’s south coast is representative of other parts of Indonesia, then the artisanal fisheries do pose a larger threat to the survival of whale sharks than previously assumed. Or in the words of Reynolds et al. [48] “Artisanal fishing pressure from small vessels (<3 m) operating in coastal waters is not captured by large, global datasets [ .. ] but may be significant in coastal waters used by tagged [whale] sharks off [ .. ] south Java, Indonesia”. Li et al. [20] reported on the landing of whale sharks in China; for the province with the highest documented number of landings, Zhejiang, during the most active period (2004–2007), around ten whale sharks were landed a year. With a coastline of over 1800 km, one and a half times that of Java’s south coast, this is markedly less than that what is landed on Java. 

The estimate of 10 to 30 whale shark landings each year along Java’s coast is a minimum one but given the species’ slow growth rate and high longevity (and consequently low natural mortality) make it highly vulnerable to overexploitation [16,17]. It is also important to note that the 19 south coastal regencies of Java are home to some 27 million people (out of 150 million on Java as a whole) and the vast majority are not involved in any way in the landing or trade of whale sharks.

### 4.2. Serendipitous Landings and Good Fortune, or Law Breaking?

Whale sharks are included on Indonesia’s protected species list, and from discussions with government officials and fishermen, this is common knowledge. Yet, whale shark landings are seen as good fortune by many. There is widespread agreement that not making good use of the whale sharks when landed is wasteful. Especially when the proceeds, or parts of the proceeds, of the whale shark parts flow back into the community by repairing roads or the local mosque, the moral principle of not profiting from legally protected species is dismissed. Elsewhere, there have been instances where the local community charged visitors money to see the landed whale sharks, with the money used to reimburse fishermen for damaged nets. Similarly, the sale of whale shark parts is also seen as a just way of compensating the fishermen in whose nets the animals accidentally ended up, as in most cases considerable damage is done to these nets. As with other protected wildlife that is sold in Pangandaran, including chambered nautilus (*Nautilus pompilius*) shells or stuffed marine turtles, the idea that the animal is already dead anyway increases the legitimization for trading these animals and their parts [32,49]. Whale sharks are not dead when they are caught, nor are marine turtles or chambered nautilus (all are actively caught often with specialised gear). This knowledge needs to be communicated wider.

While the practice of landing whale sharks is ongoing in Indonesia, what should happen when whale sharks are, deliberately or accidentally, caught? Various agreements signed by Indonesia give clear guidance. For instance, Resolution 13/05 (Conservation of whale sharks) of the WCPFC Convention requires countries to take all reasonable measures to immediately release any whale shark that has been caught accidentally. Vessels are furthermore obliged to report accidental entanglement of whale sharks and all interactions with whale sharks to the relevant authorities. As such, accidentally caught whale sharks should not be brought ashore. Furthermore, whale sharks that do get stranded, either dead or alive, should be pulled back to sea where, even after death, they have an important ecological role to play. Alternatively, where appropriate, the whale shark can be left ashore where it becomes a valuable resource for other animals (as seen in the case of the whale shark that was landed in Ngagelan, in East Java on 9 August 2022 (see Table 2) that was left in place as a food source for wildlife). There is no option in any of the international agreements that Indonesia has signed, nor in its own domestic law, to allow whale shark meat, oil, or fins to enter the trade [27]. It is evident that this is not what is happening in Pangandaran Bay or, indeed, in other parts of Java.

## 5. Conclusions

Collecting high-quality data on illicit activities is challenging, and as such, we have limited insights into illegal or unregulated fisheries. To gain better insight into this, researchers have resorted to undercover techniques to collect data [50,51], using informants embedded in the trade [52] or employing methodologies to deal with imperfect detection [53]. The research presented here suggests that using site visits where tourists congregate combined with internet searches is another way of collecting these data. Very little is known about the illegal trade in whale sharks globally [16]. While the situation with regard to whale sharks in Pangandaran Bay may be somewhat unusual, the presence of the general public at sites where illegal activities occur may not be that rare. With almost universal access to the internet and social media, monitoring illegal activities through local media and/or the general public may give greater insights into the levels of activities, their geography, and temporality [47]. When this information can be reported back to the relevant authorities in these coastal systems, this may benefit their management and their conservation. In terms of Indonesia’s performance for UN Sustainable Development Goal 14 (“Life below water”), it is on track to meeting its targets in 2030 for some of the indicators (e.g., the percentage of the total catch that comes from overexploited stock), but it is stagnant on several others (e.g., protected area coverage, clean water score, and percentage of fish caught by trawling or dredging) [9,54]. However, the present study shows that for some aspects, such as the proper implementation of measures to protect globally threatened (and legally protected) species, it falls short. It is evident that there is an urgent need to better enforce wildlife protection legislation in Indonesia, to benefit both these imperilled species and Indonesian society at large.

## Figures and Tables

**Table 1:** Details of landings of whale sharks in Pangandaran Bay, West Java, Indonesia, between July 2002 and December 2022. TL = total length (reported/measured).

Date	Location	TL (m)	Context
September 2002	Pangandaran East Beach	-/7.0	Butchered
Mid June 2009	Batu Karas	-/-	3 individuals; butchered
Early July 2009	Batu Karas	-/-	2 individuals; butchered
August 2010	Pangandaran West Beach	9/-	Butchered
September 2010	Pangandaran	8/-	[7]
21 August 2010	Pangandaran West Beach	5–6/5.0	Caught by fishermen; butchered, sold
8 January 2011	Pangandaran	15/-	Butchered, fins removed, sold
12 June 2011	Bojong Salawe Beach	6/-	Butchered, sold
30 July 2011	Pangandaran West Beach	-/-	Caught by fisherman (“happens frequently”)
2 August 2011	Parapat	-/-	Caught in nets; failed to land it
January–February 2013	Batu Hiu?	-/-	Butchered; informant from Pangandaran, June 2013, not found in media reports
11 September 2013	Bojong Salawe Beach	6.5/6.0	
30 March 2015	Pangandaran West Beach	-/8.0	
August 2015	-	-/-	Unsuccessful in pushing it back into the sea; [34]
30 November 2016	Tagog, Babakan	3; 4/3.5	Stranded and butchered
13 November 2016	Pangandaran West Beach	8/8.5	
4 December 2016	Buniayu, Karangjaladri, Parigi	5; 4–5/4.5	Butchered
January-February 2018	Pangandaran	-/-	[26]
23 August 2020	Lembah Putri, Pinggan Kalipucang	6.8; 6.88/6.5	Caught by fishermen in nets; butchered?
24 August 2020	Karangsari, Ciputrapinggan, Kalipucang	-/-	Butchered
3 March 2021	Cimanuk, Tasikmalaya	6.5/7.0	Discussions on whether local residents were allowed to butcher the animal.
5 July 2021	Batu Keras, Pangandaran	-/-	Caught by fishing line; dragged back to sea as otherwise it would be butchered
13 August 2021	East beach, Pangandaran	10/-	Stranded; dragged back to sea by fishermen
27 August 2021	Batu Hiu, Pangandaran	5/5	Stranded; dead
28 August 2021	Mandasari, Pangandaran	-/-	Stranded; dead; butchered
16 November 2021	Pamugaran, Pangandaran	-/-	Stranded; dragged back to sea
1 September 2022	East beach, Pangandaran	6/6	Stranded; dragged back to sea

**Table 2:** Whale sharks landed or stranded along Java’s south coast between August 2019 and November 2022; listed from west to east; n.a. = not available.

Location	Date	Size; Details
Wanasalam, Lebak, Banten	7 October 2019	4–5 m; left on beach
Wanasalam, Lebak, Banten	7 October 2019	n.a.; returned to sea
Muara Cidewey, Cianjur, W Java	19 September 2020	n.a.; butchered
Cipatujuh, Tasikmalaya, W Java	3 August 2020	6 m; butchered
Cimanuk, Tasikmalaya, W Java	3 March 2021	6.5 m; butchered?
Pinggan, Pangandaran, W Java	23 August 2020	7 m; buried
Batu Keras, Pangandaran, W Java	5 July 2021	n.a.; returned to sea
Batu Hiu, Pangandaran, W Java	27 August 2021	5 m; stranded, dead.
Mandasari, Pangandaran, W Java	28 August 2021	n.a.; butchered
Pamugaran, Pangandaran, W Java	16 November 2021	n.a.; dragged back to sea
Karangsari, Pangandaran, W Java	24 August 2020	n.a.; butchered
East beach, Pangandaran, W Java	13 August 2021	10 m; returned to sea
East beach, Pangandaran, W Java	1 September 2022	6 m; returned to sea
Binagung, Cilacap, C Java	13 October 2022	n.a.; butchered
Karangpakis, Cilacap, C Java	28 October 2022	6 m; butchered
Karangpakis, Cilacap, C Java	5 October 2022	15 m; butchered
Banjarsari, Cilacap, C Java	31 October 2022	6 m; butchered
Kemiren, Cilacap, C Java	6 November 2022	5 m; buried
Kertojayan, Purworejo, C Java	25 August 2020	n.a.; returned to sea
Kulon Progo, Yogyakarta	27 February 2020	n.a.; returned to sea
Kulon Progo, Yogyakarta	19 September 2020	n.a.; unknown
Kulon Progo, Yogyakarta	27 July 2022	6 m; buried
Sidomulyo, Pacitan, E Java	6 September 2022	4 m; unknown
Sidomulyo, Pacitan, E Java	6 September 2022	6 m; unknown
Paloh, Lamongan, E Java	10 September 2021	7 m; returned to sea
Paseban, Jember, E Java	30 August 2020	9 m; butchered
Nyamplong Kobong, Jember, E Java	5 July 2020	5–6 m; butchered
Nyamplong Kobong, Jember, E Java	5 July 2020	n.a.; returned to sea
Nyamplong Kobong, Jember, E Java	5 July 2020	n.a.; returned to sea
Gemukas, Jember, E Java	22 April 2021	4 m; buried
Kepanjen, Jember, E Java	15 July 2022	5 m; buried
Sumberejo, Jember, E Java	28 August 2022	6 m; buried
Paiton, Probolinggo, E Java	29 August 2019	5 m; returned to sea
Kajaran, Lumajang, E Java	9 September 2019	n.a.; buried
Bambang, Lumajang, E Java	16 September 2019	n.a.; buried
Bago, Lumajang, E Java	7 October 2019	n.a.; unknown
Wotgaluh, Lumajang, Jember	28 August 2022	n.a.; butchered?
Ngagelan, Banyuwangi, E Java	9 August 2022	4.5 m; inside national parks, left as food for wildlife

## Data Availability

All data are included in the paper; for any additional requests, please contact the corresponding author.
